# Therapeutic Effects of *Sigesbeckia pubescens* Makino Against Atopic Dermatitis-Like Skin Inflammation Through the JAK2/STAT Signaling Pathway

**DOI:** 10.3390/ijms26094191

**Published:** 2025-04-28

**Authors:** Hyun-Kyung Song, Hye Jin Kim, Seong Cheol Kim, Taesoo Kim

**Affiliations:** 1 KM Convergence Research Division, Korea Institute of Oriental Medicine, Yuseong-daero 1672, Yuseong-gu, Daejeon 34054, Republic of Korea; gusrud1654@hnibr.re.kr (H.-K.S.); kimhyejin43@kiom.re.kr (H.J.K.); iron0907@hyundaibioland.co.kr (S.C.K.); 2 Practical Research Division, Honam National Institute of Biological Resources, Gohadoan-gil 99, Mokpo 58762, Republic of Korea; 3 Biohealthcare R&D Center, Hyundai Bioland Co., Ltd., 152, Manhae-ro, Daneon-gu, Ansan 15407, Republic of Korea

**Keywords:** *Sigesbeckia pubescens* Makino, atopic dermatitis, proinflammatory chemokine, skin inflammation

## Abstract

Atopic dermatitis (AD), a chronic inflammatory skin condition, is a common allergic disorder. The human skin, the largest organ, serves as the first barrier in protecting the body against various external threats. Human epidermal keratinocytes (HEKs) in the epidermal layer and human dermal fibroblasts (HDFs) in the dermis of the skin are implicated in AD-associated skin inflammation through the secretion of diverse inflammatory mediators, including chemokines. *Sigesbeckia pubescens* Makino (SP), a traditional Korean and Chinese herbal remedy, is used for treating inflammatory conditions. While several pharmacological effects of SP extract (SPE) have been documented, its specific inhibitory effect on AD-related skin inflammation remains unexplored. Hence, oral administration of SPE to NC/Nga mice reduced the severity of house dust mite extract-induced dermatitis, accompanied by lowered levels of serum inflammatory mediators, decreased epidermal thickness, reduced mast cell infiltration, and restoration of skin barrier function within skin lesions. In conclusion, SPE has demonstrated the ability to alleviate skin inflammation and protect the skin barrier and shows potential as a therapeutic option for AD. SPE inhibited proinflammatory chemokine production by modulating the Janus kinase (JAK) 2/signal transducer and activator of transcription proteins (STAT) 1/STAT3 signaling pathway in IFN-γ- and TNF-α-stimulated skin cells.

## 1. Introduction

Atopic dermatitis (AD) is a chronic inflammatory skin condition characterized by itchy eczematous lesions [1]. Besides experiencing considerable pruritus-related distress, most patients with AD eventually develop asthma and allergic rhinitis, leading to a compromised quality of life [2]. AD results from complex interactions among immune, environmental, and genetic factors, leading to the overproduction of inflammatory mediators, including immunoglobulin (Ig) E, histamine, cytokines, and chemokines [3]. The skin characteristics of AD include increased epidermal thickness, activated T helper (Th) 2 cell immune response, mast cell infiltration, and epidermal skin barrier destruction [4,5].

The skin has two key layers—the epidermis, primarily comprising keratinocytes, which forms the protective stratum corneum barrier, and the dermis, composed of collagen and elastic tissue, which maintains skin structure and elasticity [6]. Epidermal keratinocytes and dermal fibroblasts together participate in skin inflammation, producing inflammatory mediators, including chemokines [7]. Regulating chemokine secretion is crucial for suppressing atopic inflammation in AD treatment [8]. Chemokines are signaling peptides that control immune cell infiltration and sustain inflammation in atopic skin lesions [3,9]. Most patients with AD exhibit type 2 inflammation, characterized by specific cytokines from cells such as dendritic cells, T cells, eosinophils, mast cells, and basophils [10]. Regulated upon activation, normal T-cell expressed and secreted (RANTES) and macrophage inflammatory protein (MIP)-1α act as chemo-attractants for macrophage-natural killer cells that activate T cell–dendritic cell interactions [11]. Monocyte chemo-attractant protein-1 (MCP-1) induces the recruitment of monocytes, dendritic cells, and T cells, whereas MIP-3α attracts dendritic cells and memory T cells. Eotaxin facilitates eosinophil and basophil migration, whereas thymus- and activation-regulated chemokine (TARC) contributes to the migration and activation of Th2 cells, crucial in AD [11].

*Sigesbeckia pubescens* Makino (SP) is used as an herbal remedy for inflammatory conditions in traditional Korean and Chinese medicine [12,13]. SP extract (SPE) has various pharmacological effects, including anti-allergic [14], -rheumatic [12], -inflammatory [13], -cancer [15], and -oxidant [16] effects. However, despite the recognized pharmacological activity of SPE, its potential anti-atopic effect remains poorly understood.

Therefore, in this study, we aimed to evaluate the inhibitory effect of SPE in vivo using a *Dermatophagoides farinae* (house dust mite) extract (DFE)-induced AD mouse model and interferon-γ (IFN-γ) and tumor necrosis factor-α (TNF-α)-stimulated skin cells, namely, human epidermal keratinocytes (HEKs) and human dermal fibroblasts (HDFs).

## 2. Results

### 2.1. Oral Administration of SPE Suppresses DFE-Induced AD Symptoms and Serum Inflammatory Mediator Secretion in NC/Nga Mice

**Figure 1:**
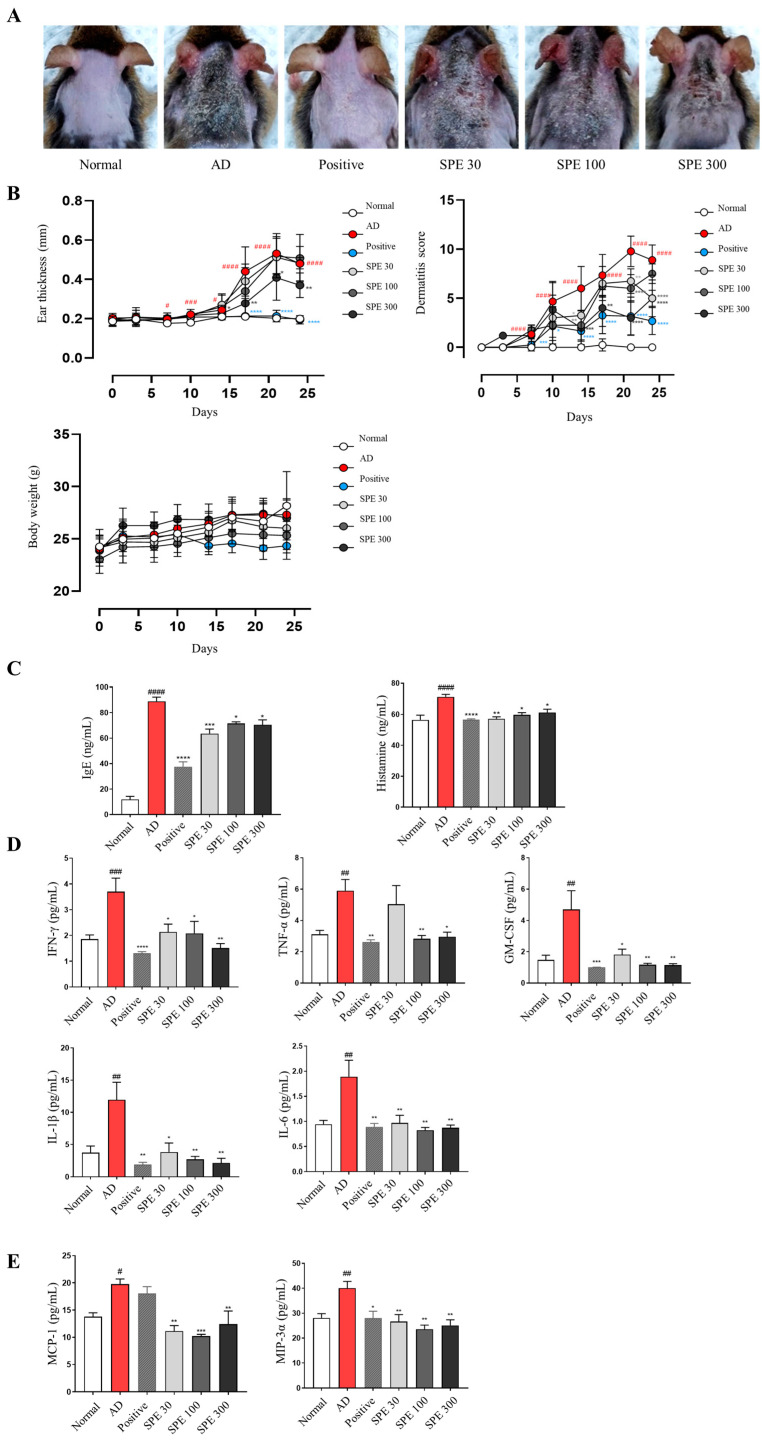
Effects of *Sigesbeckia pubescens* Makino extract (SPE) on atopic dermatitis (AD)-like symptoms and serum inflammatory mediator levels in *Dermatophagoides farinae* extract (DFE)-stimulated NC/Nga mice. Clinical symptoms on day 23 (**A**). Dermatitis score, ear thickness, and body weight were measured twice per week until the last day of the experiment (**B**). Serum immunoglobulin (Ig) E, histamine, cytokine (interferon (IFN)-γ, tumor necrosis factor (TNF)-α, granulocyte-macrophage colony-stimulating factor (GM-CSF), interleukin (IL)-1β, and IL-6), and proinflammatory chemokine (monocyte chemo-attractant protein (MCP)-1 and macrophage inflammatory protein (MIP)-3α) levels were measured using an enzyme-linked immunosorbent assay or a bead-based assay (**C**–**E**). ^#^ *p* < 0.05, ^##^ *p* < 0.005, ^###^ *p* < 0.0005, ^####^ *p* < 0.0001 vs. normal group, * *p* < 0.05, ** *p* < 0.01, *** *p* < 0.001, **** *p* < 0.0001 vs. AD group. Dexamethasone (DEXA, 1 mg/kg) was used as a positive control.

We evaluated the anti-atopic effects of SPE on DFE-induced AD-like symptoms in NC/Nga mice. The skin of the AD group exhibited maximum damage owing to DFE application, whereas SPE administration alleviated this effect (Figure 1A). In DFE-stimulated NC/Nga mice, ear thickness and dermatitis scores, measured based on edema, scarring/dryness, erythema/hemorrhage, and excoriation/erosion of AD skin lesions, were reduced upon SPE administration without any change in body weight (Figure 1B). The serum analysis revealed elevated levels of immunoglobulin E (IgE), histamine, mast cell-associated cytokines (interferon (IFN)-γ, TNF-α, granulocyte-macrophage colony-stimulating factor (GM-CSF), interleukin (IL)-1β, and IL-6), and proinflammatory chemokines (MCP-1 and MIP-3α) in the AD group. The levels of these inflammatory mediators were reduced following SPE application in DFE-stimulated NC/Nga mice (Figure 1C–E).

### 2.2. SPE Oral Administration Reduces Epidermal Thickness and Mast Cell Infiltration in DFE-Stimulated NC/Nga Mice

**Figure 2:**
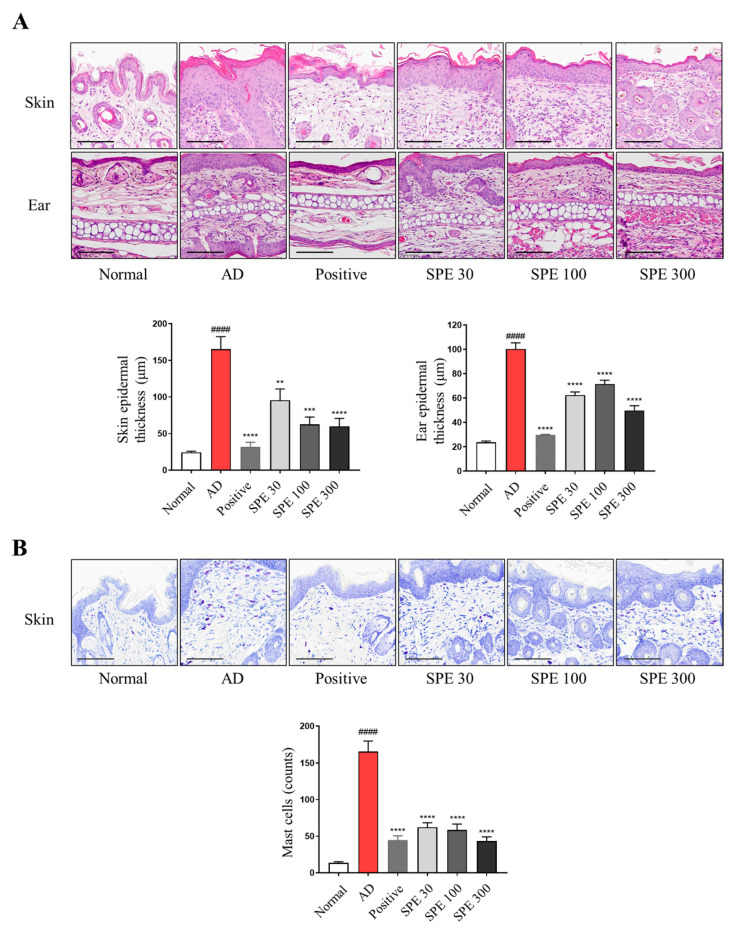
Effects of SPE on DFE-induced histological observations in NC/Nga mice. Dorsal skin and ear sections were stained with hematoxylin and eosin to confirm epidermal thickness (**A**). Skin tissue sections were stained with toluidine blue (TB) to confirm mast cell infiltration (**B**). Stained sections were visualized with a microscope at 400× magnification (scale bar = 100 µm). Data are expressed as mean ± standard error of the mean (*n* = 6). ^####^ *p* < 0.0001 vs. normal group, ** *p* < 0.01, *** *p* < 0.001, **** *p* < 0.0001 vs. atopic dermatitis (AD) group.

Increased epidermal thickness and mast cell infiltration are typical characteristics of AD lesion tissues [17]. To histologically evaluate the effect of SPE on AD-like skin and ear tissue, H&E staining and toluidine blue (TB) staining were performed. The dorsal skin and ear tissue samples from NC/Nga mice revealed a significant reduction in epidermal thickness in the SPE group (Figure 2A). TB staining confirmed that DFE-induced mast cell infiltration decreased following SPE administration (Figure 2B). 

### 2.3. SPE Oral Administration Attenuates DFE-Induced Changes in Skin Barrier-Related Protein Levels in NC/Nga Mouse Skin Lesions

**Figure 3:**
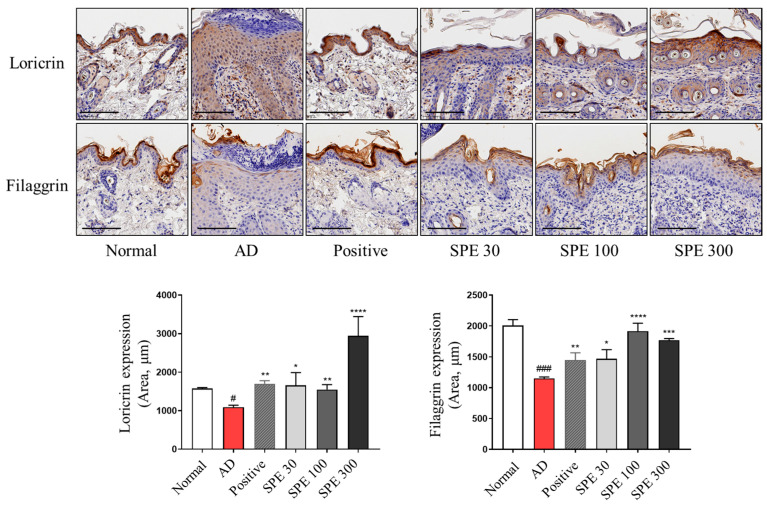
Effects of SPE on skin barrier dysfunction induced using DFE in NC/Nga mice. Paraffin-embedded skin tissues were immunostained with loricrin and filaggrin antibodies (scale bar = 100 µm). Quantification of dark brown regions was performed to assess loricrin and filaggrin expression. Data are presented as mean ± standard error of the mean (*n* = 6). ^#^ *p* < 0.05, ^###^ *p* < 0.001 vs. normal group, * *p* < 0.05, ** *p* < 0.01, *** *p* < 0.001, **** *p* < 0.0001 vs. atopic dermatitis (AD) group.

In patients with AD, the expression of skin barrier-related proteins was reportedly decreased [4,18]. Moreover, in AD progression, the skin barrier compromised because of allergies is thought to play a role in prompting scratching behavior [4]. Therefore, the expression of skin barrier-related proteins, such as loricrin and filaggrin, was examined using immunohistochemistry. As shown in Figure 3, topical administration of SPE reversed the DFE-induced attenuation of loricrin and filaggrin expression in the skin tissue. These results suggest that the application of DFE to NC/Nga mouse skin could lead to skin barrier dysfunction, which can be prevented by SPE.

### 2.4. SPE Inhibits Proinflammatory Chemokine Production in IFN-γ/TNF-α-Stimulated Skin Cells

**Figure 4:**
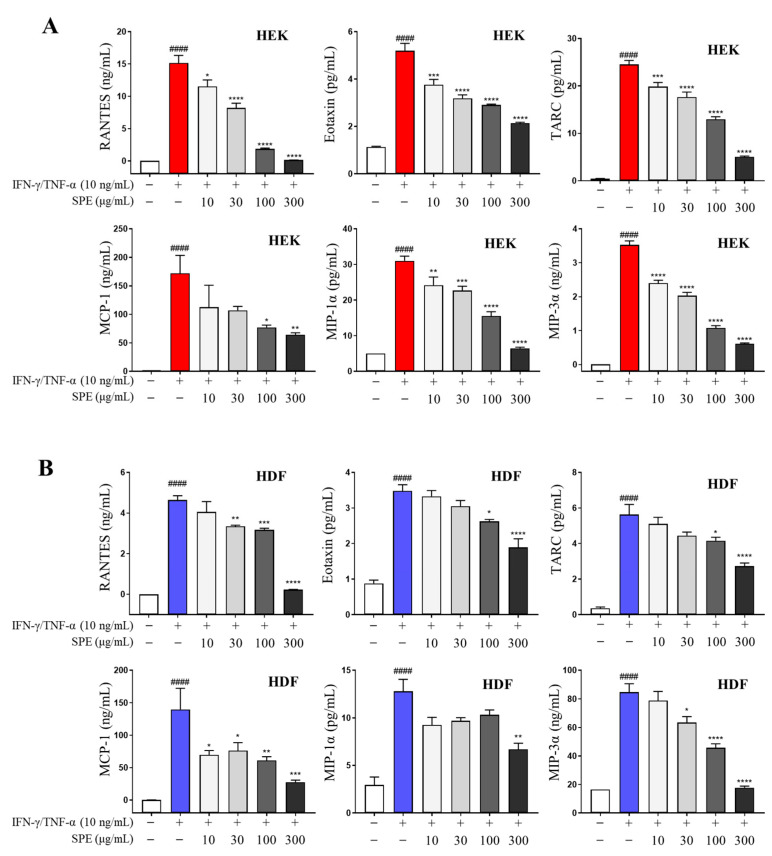
Effects of SPE on IFN-γ- and TNF-α-induced proinflammatory chemokine production in skin cells. Cells were pretreated with SPE for 1 h and then stimulated with 10 ng/ml of IFN-γ/TNF-α for 24 h. Proinflammatory chemokine secretion was monitored using a bead-based assay (**A**,**B**). Data are presented as mean ± standard error of the mean (*n* = 4). ^####^ *p* < 0.0001 vs. untreated; * *p* < 0.05, ** *p* < 0.01, *** *p* < 0.001, **** *p* < 0.0001 vs. IFN-γ/TNF-α. HEKs, human epidermal keratinocytes; RANTES, regulated upon activation, normal T cell expressed and secreted; TARC, thymus- and activation-regulated chemokine; MCP-1, monocyte chemo-attractant protein-1; MIP-1α, macrophage inflammatory protein 1α; HDFs, human dermal fibroblasts.

The Cell Counting Kit-8 (CCK-8) assay revealed that 24 h treatment with SPE at concentrations ranging from 0 to 300 μg/mL did not significantly affect HEK and HDF viability (Supplementary Figure S1). Therefore, the skin cells were treated with SPE at concentrations of ≤300 μg/mL in the subsequent experiments. We investigated the effect of SPE on the production of IFN-γ/TNF-α-induced inflammatory chemokines such as RANTES, eotaxin, TARC, MCP-1, MIP-1α, and MIP-3α. SPE effectively inhibited proinflammatory chemokine production in the skin cells (Figure 4).

### 2.5. SPE Inhibits IFN-γ/TNF-α-Induced JAK2/STAT Activation in Skin Cells

**Figure 5:**
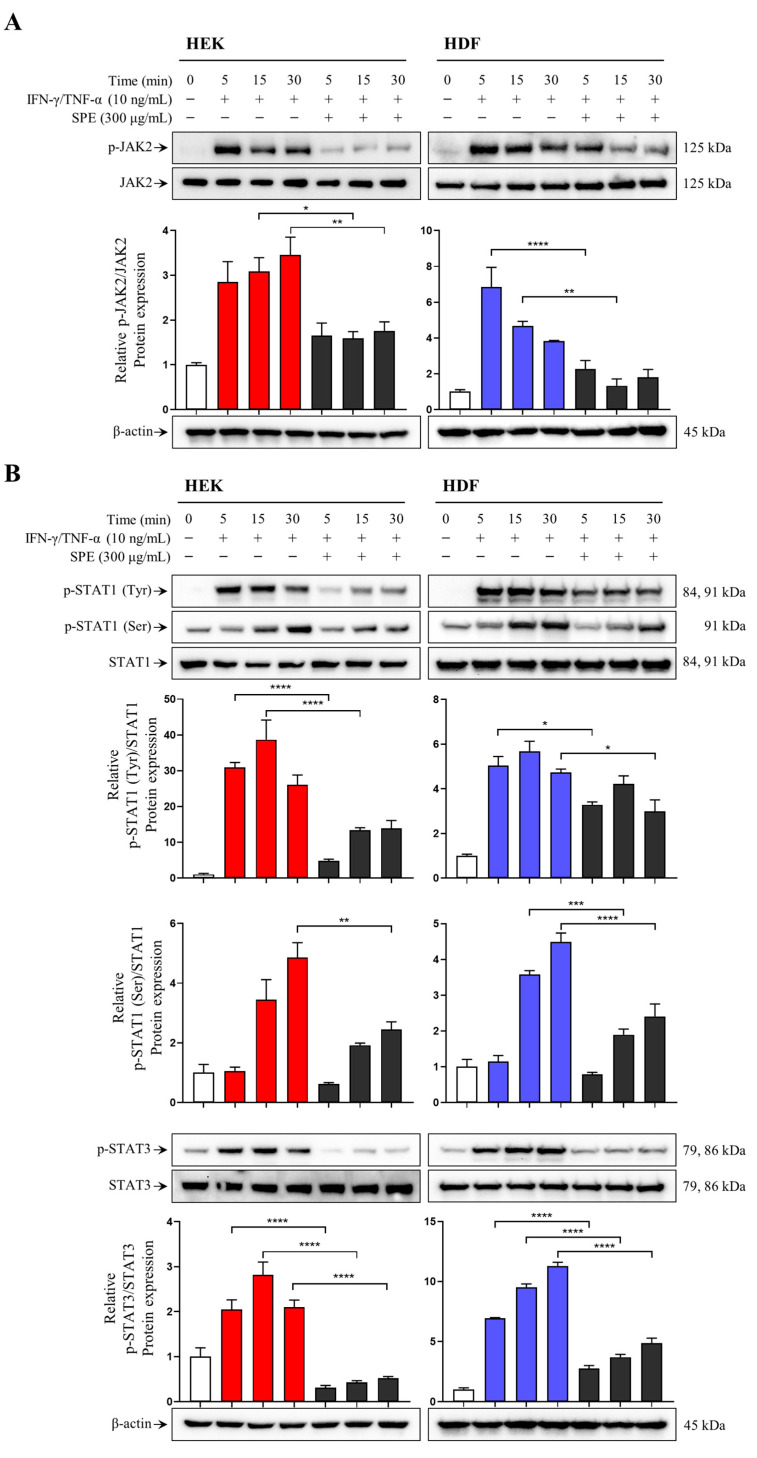
Effects of SPE on IFN-γ- and TNF-α-induced Janus kinase (JAK)2/signal transducer and activator of transcription (STAT) activation in skin cells. The cells were pretreated with SPE for 1 h and then stimulated with 10 ng/ml of IFN-γ/TNF-α for various time intervals (0, 5, 15, and 30 min). Expression of total and phosphorylated JAK2 (**A**) as well as STAT1 and STAT3 (**B**) in the total lysate of HEKs and HDFs was measured using Western blotting. β-actin was used as a loading control. Blots are representative of three independent experiments with similar results. Quantification data of protein are presented as mean ± standard error of the mean (*n* = 3). * *p* < 0.05, ** *p* < 0.01, *** *p* < 0.001, **** *p* < 0.0001 vs. each time point of the IFN-γ/TNF-α group.

JAK/STAT signaling plays a pivotal role in immune response, including that of AD [19]. Therefore, we investigated the effects of SPE on JAK2 and STAT1/3 activation in skin cells using Western blotting. IFN-γ/TNF-α treatment induced JAK2 phosphorylation, which was effectively suppressed by SPE in HEKs and HDFs (Figure 5A). We also assessed the effect of SPE on transcription factors such as STAT1 and STAT3. SPE treatment significantly inhibited STAT1 and STAT3 phosphorylation (Figure 5B). SPE regulated IFN-γ/TNF-α-induced proinflammatory chemokine secretion through the modulation of JAK2/STAT activation.

### 2.6. SPE Inhibits IFN-γ/TNF-α-Induced STAT1/3 Translocation in Skin Cells

**Figure 6:**
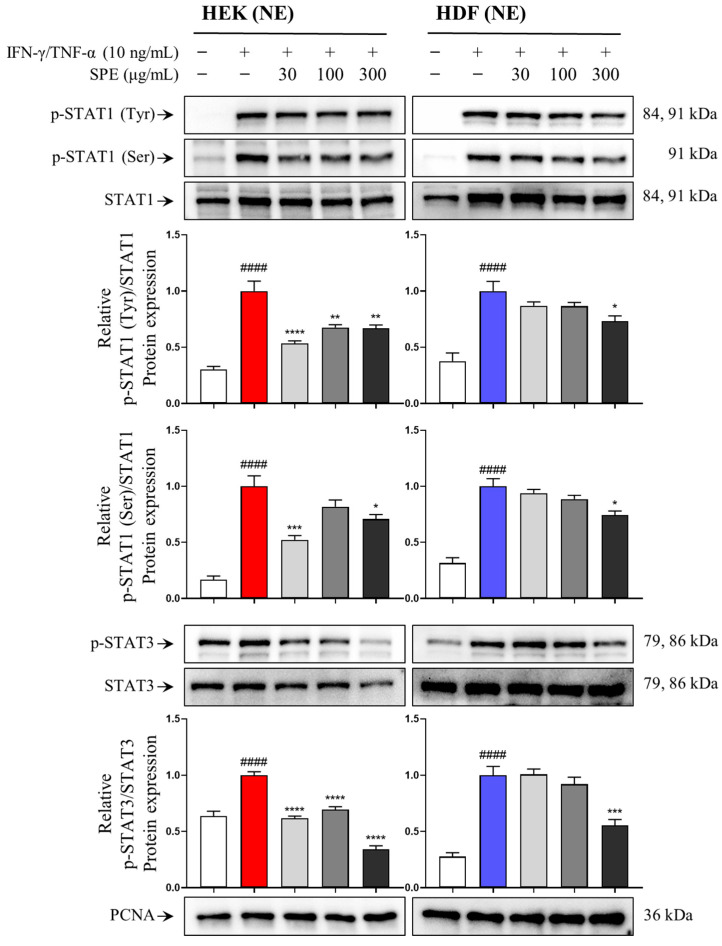
Effects of SPE on IFN-γ- and TNF-α-induced STAT translocation in skin cells. Cells were pretreated with SPE for 1 h and then stimulated with 10 ng/ml of IFN-γ/TNF-α for 3 h. Nuclear proteins were isolated, and the total and p-STAT1 and p-STAT3 nuclear translocation levels were determined using Western blotting. PCNA was used as a loading control for nuclear extracts (NEs). Blots are representative of three independent experiments with similar results. Quantification data of protein are presented as mean ± standard error of the mean (*n* = 3). ^####^ *p* < 0.0001 vs. untreated; * *p* < 0.05, ** *p* < 0.01, *** *p* < 0.001, **** *p* < 0.0001 vs. IFN-γ/TNF-α.

We previously showed that SPE inhibits the activation of STAT1 and STAT3 in response to IFN-γ/TNF-α in HEKs and HDFs. When STAT1 and STAT3 are activated, they move from the cytoplasm to the nucleus, where they bind to genes that trigger the production of inflammatory chemokines [20]. Therefore, we investigated the effect of SPE on the nuclear translocation of phosphorylated STAT1 and STAT3 in IFN-γ/TNF-α-stimulated HEKs and HDFs using nuclear fractions and Western blotting. SPE pretreatment dose-dependently inhibited the translocation of phosphorylated STAT1 and STAT3 (Figure 6).

### 2.7. High-Performance Liquid Chromatography (HPLC) of SPE

In the HPLC analysis of SPE, kirenol was detected at 215 nm, whereas five compounds (chlorogenic acid, rutin, isoquercitrin, quercitrin, and quercetin) were detected at 254 nm. All compounds were separated within 35 min, and the retention time of chlorogenic acid, rutin, isoquercitrin, quercitrin, quercetin, and kirenol was 4.71, 9.05, 11.57, 16.36, 26.94, and 28.80 min, respectively (Supplementary Figure S2). Calibration curves were used to quantitatively analyze these six compounds, all of which had regression equations with a coefficient of determination (r^2^) of ≥0.993. The level of chlorogenic acid was higher (1.27%) than that of rutin, isoquercitrin, quercitrin, quercetin, and kirenol (0.21%, 0.17%, 0.59%, 0.03%, and 0.58%, respectively) (Table 1).

### 2.8. SPE Constituents Inhibit Proinflammatory Chemokine Production in IFN-γ/TNF-α-Stimulated Skin Cells

We validated the effects of chlorogenic acid, kirenol, and quercitrin, the major compounds identified following HPLC analysis of SPE, on proinflammatory chemokine production in IFN-γ/TNF-α-induced skin cells. To determine the experimental concentrations of the three compounds, their cytotoxicity was assessed (Supplementary Figure S3). Consequently, cells were treated with 0–100 µg/mL of the compounds in the subsequent experiments.

**Figure 7:**
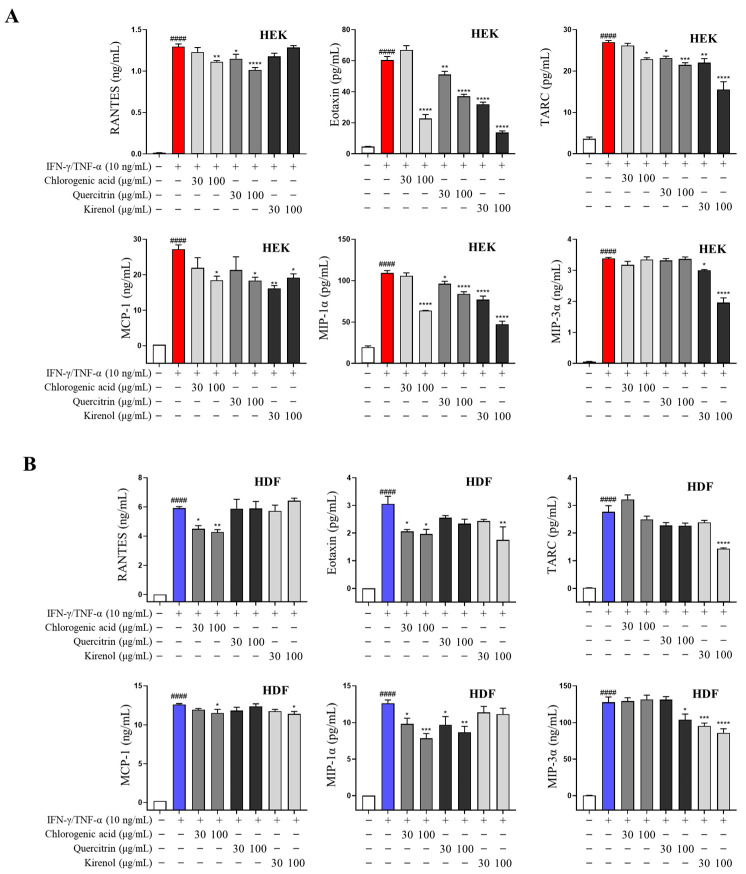
Effects of SPE constituents on IFN-γ- and TNF-α-induced proinflammatory chemokine production in skin cells. Proinflammatory chemokine secretion was confirmed using a bead-based assay (**A**,**B**). Data are presented as mean ± standard error of the mean (*n* = 4). ^####^ *p* < 0.0001 vs. untreated; * *p* < 0.05, ** *p* < 0.01, *** *p* < 0.001, **** *p* < 0.0001 vs. IFN-γ/TNF-α.

Chlorogenic acid, kirenol, and quercitrin exerted potent anti-inflammatory effects by significantly suppressing proinflammatory chemokine production in IFN-γ/TNF-α-induced skin cells (Figure 7). The anti-inflammatory effects of SPE are likely attributable to the presence of active compounds such as chlorogenic acid, kirenol, and quercitrin, which have been reported to exert anti-inflammatory and anti-AD activities. These compounds may contribute to the observed suppression of AD-associated inflammation.

## 3. Discussion

Traditionally known as the “rheumatism herbs” in China, the annual herbs of the genus *Sigesbeckia* have been used as an herbal medicine to control inflammation [12]. In the 2015 edition of the Chinese Pharmacopoeia, three species of the *Sigesbeckia* genus were introduced, namely, *S. glabrescens* Makino, *S. orientalis* L., and SP [21]. Of these species, SP was selected as the focus of our study. This plant is native to East Asia, particularly Korea, Japan, and China, and it grows naturally in mountainous and field regions [22]. Traditionally, it is harvested during its mature flowering period for medicinal purposes [23]. SP reportedly exhibits various physiological activities. It plays a role in the prevention and treatment of cardiovascular and cerebrovascular diseases [24], joint and neurological disorders [25,26], and ulcerative colitis [27]. SP also has strong antioxidant [16], anti-bacterial [25,28], anti-tumor [29], and anti-inflammatory [22,30,31] properties.

Mast cells are key effector cells in IgE-mediated allergic diseases, particularly those associated with pruritus [1]. IgE binding to mast cells leads to the release of histamine and inflammatory cytokines, including IFN-γ, TNF-α, GM-CSF, IL-1β, and IL-6 [32]. Histamine from mast cells is a potent inducer of pruritus, a hallmark of AD [32]. Thus, regulating mast cell infiltration and activation is crucial in alleviating AD-related itching symptoms. In the present study, mast cell infiltration was observed in DFE-stimulated NC/Nga mice, and it was effectively mitigated by SPE application.

Oral administration of SPE reduced serum levels of IgE, histamine, and mast cell-related cytokines. The observed increase in epidermal thickness, a characteristic feature of AD, was also alleviated by SPE, as evidenced by H&E tissue staining. Overall, SPE administration alleviated AD-like symptoms, including increased ear thickness and dermatitis scores. Epidermal skin barrier dysfunction and inflammation play key roles in AD development [4]. Moreover, in the course of AD development, the repetitive scratching behavior, often associated with allergies, is implicated in skin barrier disruption [4]. Structural proteins within the epidermis, including loricrin and filaggrin, are pivotal for the formation of the epidermal skin barrier [18]. In our study, in the SPE-treated group, protection against skin barrier destruction induced by AD was observed.

We speculated that SPE prevented AD development by regulating the JAK/STAT pathway, which is involved in the regulation of various inflammatory factor levels, including proinflammatory chemokines, and contributes to the regulation of various immune responses [33]. JAK/STAT plays a crucial role in regulating Th2 cell differentiation in AD, a Th2-dominant disease [34]. JAK2 inhibition suppresses STAT1 and STAT3 activation and tissue inflammation, including that of the skin [35]. STAT1 activation under IFN-γ/TNF-α stimulation is involved in the regulation of Th2-related chemokine levels, such as RANTES and TARC [17]. Concordantly, IFN-γ/TNF-α stimulation increased JAK2, STAT1, and STAT3 phosphorylation in HEKs and HDFs, whereas SPE treatment substantially counteracted these effects. Overall, STAT is a transcription factor that activates target genes by translocating to the nucleus and binding to regulatory elements in gene promoters [36]. In the present study, we evaluated the inhibitory effects of SPE on IFN-γ/TNF-α-induced nuclear translocation of STAT by confirming the levels of phosphorylated STAT1 and STAT3 in nuclear proteins isolated from skin cells. 

Recent studies have reported that *Siegesbeckiae Herba* contains various flavonoids [37], which exhibit a range of beneficial pharmacological activities. In line with these findings, our phytochemical analysis of the extracts used in this study identified several flavonoid compounds, including chlorogenic acid, rutin, isoquercitrin, quercitrin, quercetin, and kirenol. The most abundant compound in SPE, chlorogenic acid, alleviates the symptoms of allergic rhinitis and asthma [38]. Although reports on the effectiveness of kirenol against allergic diseases are lacking, it has been shown to exhibit anti-inflammatory and analgesic activities [39]. Another major component, quercitrin, reportedly exerts anti-inflammatory [40] and anti-cancer [41] effects and inhibits contact dermatitis development [42]. Our results confirmed that chlorogenic acid, quercitrin, and kirenol inhibit the expression of inflammatory chemokines induced by IFN-γ/TNF-α in skin cells. Therefore, the anti-atopic effects of SPE may result from synergy among these components.

## 4. Materials and Methods

### 4.1. Preparation of SPE

Dried SP was refluxed in 70% ethanol for 3 h at a controlled temperature of 100 °C ± 2 °C. The obtained SPE was evaporated at a low temperature after filtration using a 53 μm mesh. SPE was then acquired by removing the solvent using a rotary evaporator, followed by freeze-drying; the resulting SPE powder was stored at −20 °C. For in vitro experiments, SPE was dissolved in dimethyl sulfoxide (DMSO) and passed through a 0.2-µm polytetrafluoroethylene syringe filter (Corning Inc., Corning, NY, USA). For in vivo investigations, the SPE powder was dissolved in water and orally administered to mice. The plant name has been verified at http://www.theplantlist.org (accessed on 1 January 2024).

### 4.2. Animals

Eight-week-old male NC/Nga mice (body weight, 19–24 g) were sourced from Central Lab Animal, Inc. (Seoul, Republic of Korea). The sex of our animals for this model did not influence the research outcomes. The mice were maintained under controlled standard conditions following the guidelines of the Institutional Animal Care and Use Committee of the Korea Institute of Oriental Medicine. The experimental protocols were approved by the concerned ethics committee (Approval No. 210-089). NC/Nga mice were housed in groups of six per cage and allowed to acclimatize to the animal room for 1 week. The mice were randomly divided into six groups, each consisting of four mice.

### 4.3. AD Induction and Symptom Analysis

To sensitize the skin barrier, we applied 200 µL of 4% sodium dodecyl sulfate (SDS) onto the shaved dorsal skin and ear lesions of NC/Nga mice. NC/Nga mice, which spontaneously develop AD-like skin lesions under conventional housing conditions, are widely used as a validated animal model for studying atopic dermatitis due to their similarity to human AD symptoms [43,44]. After 1 h, we administered 100 mg of DFE ointment (Biostir Inc., Kobe, Japan) onto the dorsal skin and ears. AD was induced twice weekly for 3 weeks. On day 3 of AD induction, SPE or DEXA (1 mg/kg) was orally administered daily until the mice were sacrificed. Dermatitis score, ear thickness, and body weight were recorded twice per week. The dermatitis score was evaluated based on the sum of the severity index scores (0, no; 1, mild; 2, moderate; 3, severe), considering parameters such as edema, scarring/dryness, erythema/hemorrhage, and excoriation/erosion [45]. Ear thickness was measured using a digital caliper (CAS ©, Seoul, Republic of Korea) at the same site when possible, using a single caliper to minimize variations.

### 4.4. Serum Analysis

Blood was collected from the sacrificed mice. Serum samples were obtained by centrifuging the blood at 3000 rpm for 15 min and stored at –80 °C until further use. Using the LBIS Mouse IgE ELISA Kit (Fujifilm, Shibukawa, Japan) and Histamine Research ELISATM (LDN, Nordhorn, Germany), we determined total IgE and histamine levels in the serum using an enzyme-linked immunosorbent assay (ELISA) following the manufacturer’s instructions. The serum levels of proinflammatory chemokines (MCP-1 and MIP-3) and cytokines (IFN-γ, TNF-α, GM-CSF, IL-1β, and IL-6) were assessed using a bead-based assay with the LEGENDplex™ Mouse Proinflammatory Chemokine Panel or Inflammation Panel (BioLegend, San Diego, CA, USA) following the manufacturer’s protocol.

### 4.5. Histological Observation and Immunohistochemistry

For histological evaluation, the excised mouse dorsal back skin and ears were fixed in 10% formaldehyde and embedded in paraffin. The tissues were stained with hematoxylin and eosin (H&E) staining solution or TB solution (Sigma-Aldrich, St. Louis, MO, USA). For immunohistochemistry, the slide-mounted tissue samples were incubated overnight at 4 °C with loricrin (Abcam, Cambridge, UK) and filaggrin (Enzo, NY, USA) antibodies. The stained tissue samples were scanned and analyzed using an automatic digital slide scanning system (Kfbio, Ningbo, China).

### 4.6. Cells and Reagents

Neonatal HEKs were cultured in Medium 154 supplemented with human keratinocyte growth supplement, 10% fetal bovine serum (FBS), and 1% penicillin/streptomycin (PS). Neonatal HDFs were cultured in Dulbecco’s modified Eagle’s medium supplemented with 10% FBS and 1% PS. All culture media, supplements, and phosphate-buffered saline (PBS) were procured from Gibco BRL (Gaithersburg, MD, USA). HEKs and HDFs were cultured in an incubator at 37 °C under 5% CO_2_. Recombinant human IFN-γ and TNF-α were obtained from Thermo Fisher Scientific (Waltham, MA, USA). DMSO, SDS, DEXA, and skim milk were purchased from Sigma-Aldrich. Proliferating cell nuclear antigen and horseradish peroxidase-conjugated (HRP) secondary antibodies were procured from Santa Cruz Biotechnology (Santa Cruz, CA, USA); antibodies against p-Janus kinase (JAK) 2, JAK2, p-signal transducer and activator of transcription (STAT)1 (Tyr), p-STAT1 (Ser), STAT1, p-STAT3, STAT3, and β-actin were obtained from Cell Signaling Technology (Beverly, MA, USA).

### 4.7. Cell Viability

The viability of HEKs and HDFs was determined using the CCK-8 (Dojindo Molecular Technologies Inc., Rockville, MD, USA). HEKs (2 × 10^4^ cells/well) and HDFs (5 × 10^3^ cells/well) were seeded in 96-well plates. After 24 h, the cells were treated with various concentrations of SPE (0–300 µg/mL) and incubated for 24 h. CCK-8 reagent (10 µL) was added to each well, and the samples were incubated for 2 h at 37 °C. Absorbance of the samples at 450 nm was determined using a SpectraMax 340 microplate reader (Molecular Devices, CA, USA).

### 4.8. Proinflammatory Chemokine Bead-Based Assay

The proinflammatory chemokine levels were determined using the LEGENDplex™ Proinflammatory Chemokine panel (BioLegend), following the manufacturer’s protocol. The assay was conducted on a BD LSRFortessa™ Flow Cytometer (BD Biosciences, San Jose, CA, USA) using the BD CellQuest™ software. Data were analyzed using the LEGENDplex™ v8.0 software (VigeneTech Inc., Carlisle, MA, USA).

### 4.9. Western Blotting

HEKs and HDFs, seeded at 80–90% density, were stimulated with IFN-γ/TNF-α at the indicated concentrations. Whole-cell lysates were prepared using PRO-PREP extraction buffer (Intron, Seoul, Republic of Korea). Protein (10 µg) was separated using 10% Mini-PROTEAN TGX Precast Protein Gels (Bio-Rad, Hercules, CA, USA) and transferred onto Hybond™ polyvinylidene fluoride membranes (GE Healthcare Life Sciences, Little Chalfont, UK). The membranes were blocked using 5% skim milk or bovine serum albumin (MP Biomedicals, Irvine, CA, USA) in Tris-buffered saline with 1% Tween-20 (TBST) for 2 h and then incubated overnight with primary antibodies diluted at 1:1000 at 4 °C with gentle agitation. After washing with TBST, the membranes were incubated with HRP-conjugated secondary antibodies for 1 h. Protein bands were visualized and analyzed using the ChemiDoc Imaging System (Bio-Rad).

### 4.10. Nuclear Fraction Analysis

To isolate the nuclear fraction, we employed NE-PER^®^ Nuclear and Cytoplasmic Extraction Reagents (Pierce Biotechnology, Rockford, IL, USA). The cells were rinsed with cold PBS, harvested, and lysed in CER buffer with Halt™ Protease and Phosphatase Inhibitor Cocktail (Thermo Fisher Scientific) on ice for 10 min. After centrifugation at 14,000 rpm for 5 min, the supernatants were discarded, and the cell pellets were lysed in NER buffer with the inhibitor cocktail for 40 min on ice. Following centrifugation at 14,000 rpm for 10 min, the resulting supernatants, containing nuclear proteins, were collected. Protein levels were assessed using Western blotting.

### 4.11. HPLC

HPLC was performed using a Shimadzu LC-20A System equipped with an SPD-M20A Prominence diode array detector (Shimadzu, Kyoto, Japan) set at detection wavelengths of 215 and 254 nm. Separation was performed on a Phenomenex Luna C18 column (250 mm × 4.6 mm; particle size 5 μm; Phenomenex, Torrance, CA, USA) at 25 °C with a gradient mobile phase comprising 0.1% aqueous formic acid (A) and 0.1% formic acid in acetonitrile (B). The gradient conditions were as follows: 0–5 min, 0–20% B; 5–30 min, 20–35% B; and 30–35 min, 35–50% B. The flow rate was 1.0 mL/min, with an injection volume of 10 μL per sample. For quantitative analysis, SPE was dissolved in methanol (10 mg/mL) via sonication for 15 min and subsequently passed through a 0.22 μm syringe filter. The six reference compounds used in the analysis were procured from Chemfaces (Wuhan, China); their purity was ≥98%.

### 4.12. Statistical Analysis

Data are presented as mean ± standard error of the mean. Multiple group comparisons were performed using a one-way analysis of variance followed by Tukey’s test. GraphPad Prism (version 8.0; GraphPad Software, Inc., San Diego, CA, USA) was employed for statistical analysis. Statistical significance was set at *p* < 0.05.

## 5. Conclusions

Topical application of SPE decreased the development of AD-like skin inflammation in DFE-treated NC/Nga mice and IFN-γ/TNF-α-treated skin cells. In vitro, SPE suppressed JAK2/STAT1/STAT3 signaling in the skin cells, and this effect is attributable to its constituents, including chlorogenic acid, quercitrin, and kirenol. These findings suggest that SPE holds promise as a potential alternative therapy for managing AD, offering a targeted approach to alleviate inflammation while minimizing potential side effects commonly associated with conventional treatments.

## Figures and Tables

**Table 1:** Calibration curve data and content of chemical compounds in *Sigesbeckia pubescens* Makino extract.

Peak No.	Compound	Regression Equation				Content (%)
		Linear Range (μM)	Slope	Intercept	r^2^	
1	Chlorogenic acid	6.25–100	12,054	−54,195	0.993	1.27
2	Rutin	6.25–100	30,227	−117,870	0.994	0.21
3	Isoquercitrin	6.25–100	20,066	−82,796	0.994	0.17
4	Quercitrin	6.25–100	23,199	−98,109	0.994	0.59
5	Quercetin	6.25–100	44,099	−199,847	0.995	0.03
6	Kirenol	6.25–100	8057	−31,424	0.993	0.58

## Data Availability

Data will be made available on request.

## Supplementary Materials

The following supporting information can be downloaded at: https://www.mdpi.com/article/10.3390/ijms26094191/s1.

## Abbreviations

AD	atopic dermatitis
DFE	*Dermatophagoides farinae* extract
FBS	fetal bovine serum
H&E	hematoxylin and eosin
HDF	human dermal fibroblast
HEK	human epidermal keratinocyte
HKGS	human keratinocyte growth supplement
HRP	horseradish peroxidase
IFN-γ	interferon-γ
Ig	immunoglobulin
JAK	Janus kinase
PBS	phosphate-buffered saline
SEM	standard error of the mean
SPE	*Sigesbeckia pubescens* Makino extract
STAT	signal transducer and activator of transcription proteins
TB	toluidine blue
Th	T helper
TNF-α	tumor necrosis factor-α
