# Supplementary material for: Therapeutic Effects of Sigesbeckia pubescens Makino Against Atopic Dermatitis-Like Skin Inflammation Through the JAK2/STAT Signaling Pathway
Source: Int J Mol Sci. 2025 Apr 28;26(9):4191. doi: 10.3390/ijms26094191 (PMC12071371; doi:10.3390/ijms26094191)
Supplement: Supplementary file 1 [file ijms-26-04191-s001.zip › ijms-3601667-supplementary.pdf]

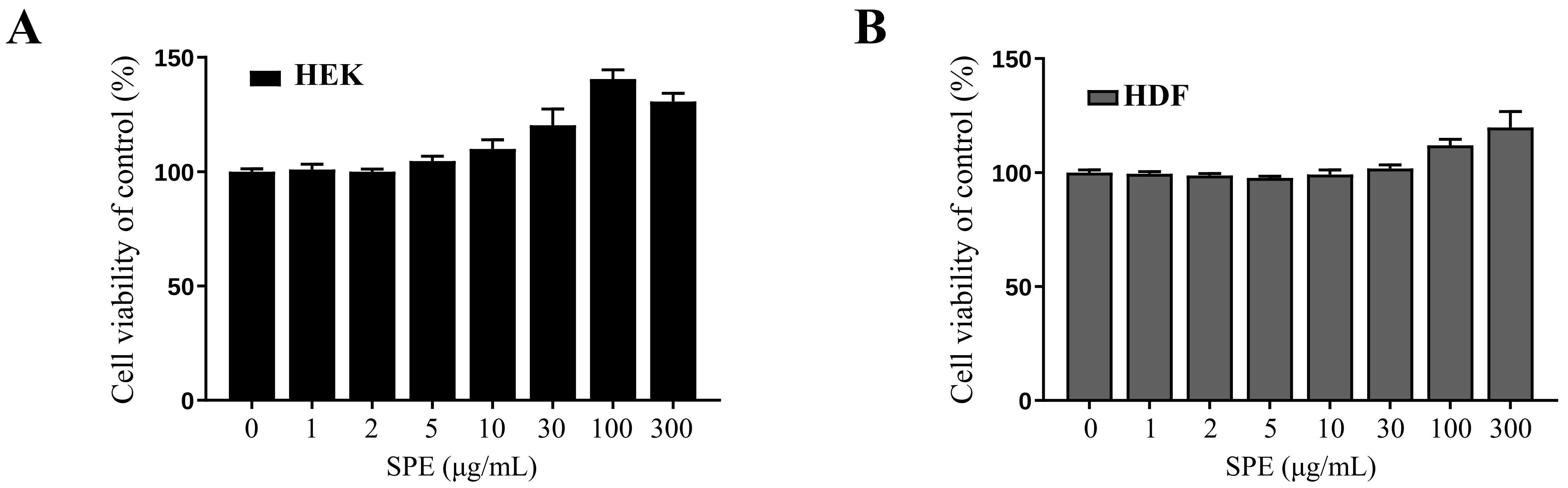

Supplementary figure 1. Effects of *Sigesbeckia pubescens* Makino extract (SPE) on cell viability were assessed using the Cell Counting Kit-8 assay after 24 h of incubation (A, B).

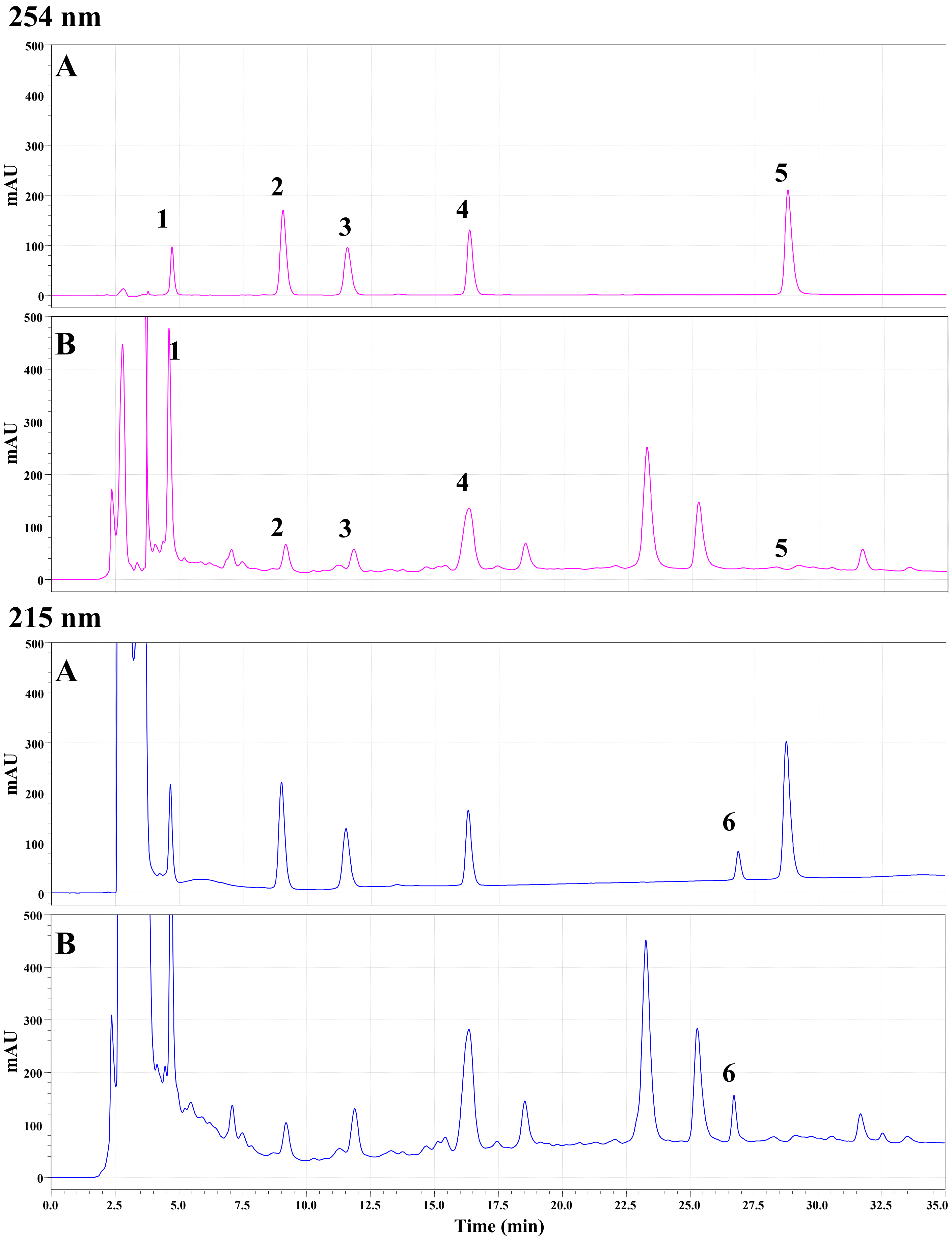

Supplementary figure 2. High-performance liquid chromatography analysis of the standard mixture (A) and SPE (B) at 254 and 215 nm, respectively. 1, chlorogenic acid; 2, rutin; 3, isoquercitrin; 4, quercitrin; 5, quercetin; 6, kirenol.

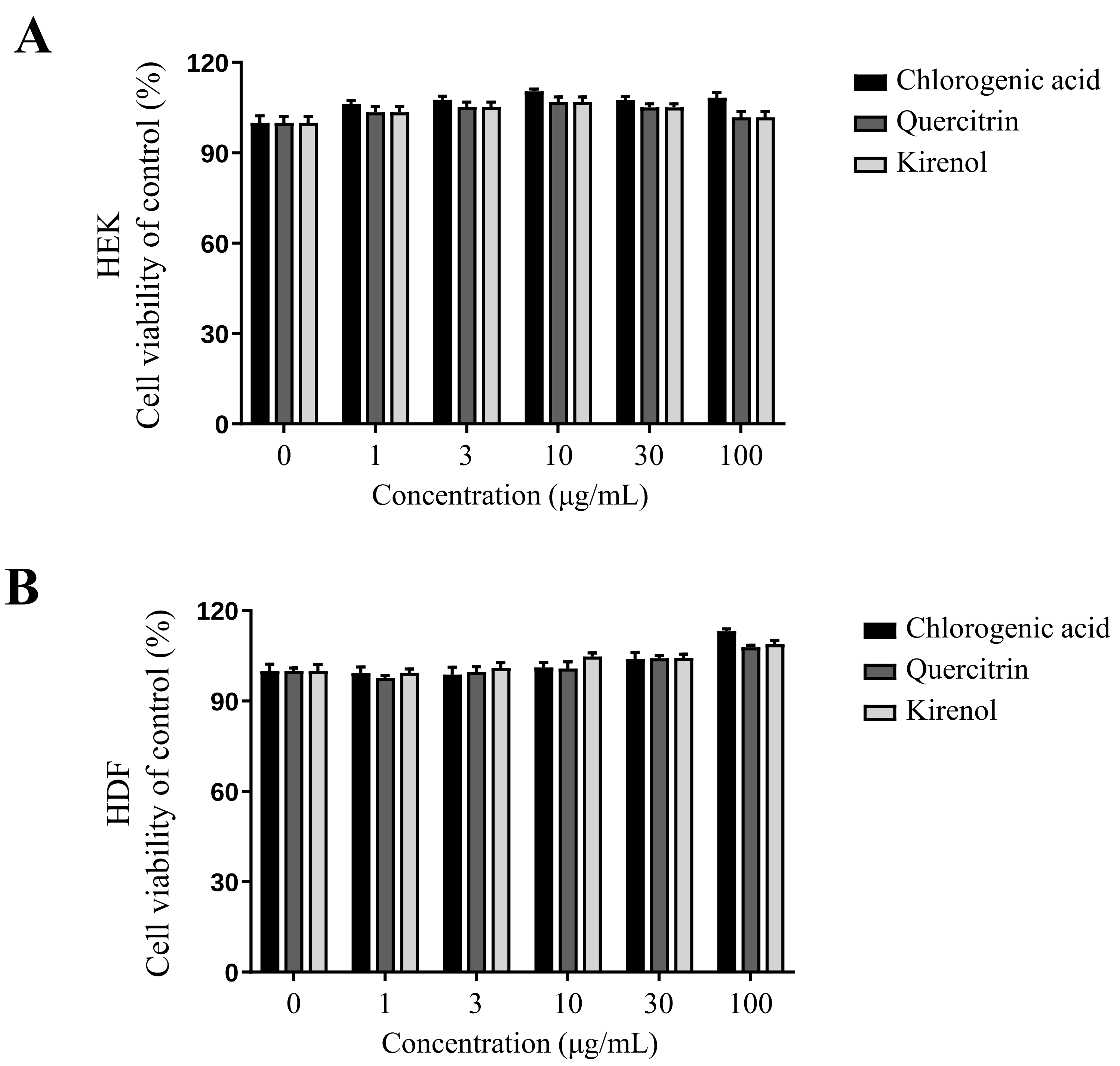

Supplementary figure 3. Effects of the compounds on cell viability were assessed after 24 h of incubation (A, B).
